# Residency Training in Robotic General Surgery: A Survey of Program Directors

**DOI:** 10.1155/2018/8464298

**Published:** 2018-05-08

**Authors:** Lea C. George, Rebecca O'Neill, Aziz M. Merchant

**Affiliations:** Department of Surgery, Rutgers New Jersey Medical School, Newark, NJ 07103, USA

## Abstract

**Objective:**

Robotic surgery continues to expand in minimally invasive surgery; however, the literature is insufficient to understand the current training process for general surgery residents. Therefore, the objectives of this study were to identify the current approach to and perspectives on robotic surgery training.

**Methods:**

An electronic survey was distributed to general surgery program directors identified by the Accreditation Council for Graduate Medical Education website. Multiple choice and open-ended questions regarding current practices and opinions on robotic surgery training in general surgery residency programs were used.

**Results:**

20 program directors were surveyed, a majority being from medium-sized programs (4–7 graduating residents per year). Most respondents (73.68%) had a formal robotic surgery curriculum at their institution, with 63.16% incorporating simulation training. Approximately half of the respondents believe that more time should be dedicated to robotic surgery training (52.63%), with simulation training prior to console use (84.21%). About two-thirds of the respondents (63.16%) believe that a formal robotic surgery curriculum should be established as a part of general surgery residency, with more than half believing that exposure should occur in postgraduate year one (55%).

**Conclusion:**

A formal robotics curriculum with simulation training and early surgical exposure for general surgery residents should be given consideration in surgical residency training.

## 1. Introduction

Since its emergence, robotic surgery technology has seen rapid global adoption across many surgical disciplines including urology, gynecology, and general surgery and, now, robotic surgery is a mainstay of minimally invasive surgery in the United States [1–4]. The growth of robotics into general surgery is partially due to its continuous advancements, such as enhanced visual control and quality, improved manipulation of instruments, and elimination of tremor [1, 5–7]. However, despite the growing field of robotic surgery, there have been only minor changes in the general surgery residency curriculum to incorporate robotic surgery education [8].

In the first few years following robotic surgery FDA approval, several studies explored methods of implementing robotic surgery into general surgery residency training. In 2002, Donias et al. found that only 23% of responding general surgery program directors wished to incorporate robotic training into their residency programs [9]. A year later, Patel et al. discovered that 57% of residents had a strong interest in robotic surgery, yet 80% of them did not have an established robotics training program at their institution [10]. More recently, based on a survey study from 2013, Farivar et al. uncovered that while 96% of US residents have a surgical robot system at their institution, only 63% of residents have participated in a robotic case. Furthermore, 60% of those residents did not receive any robotics training prior to participation [5].

Since these studies, the use of the surgical robot in general surgery has expanded significantly, along with an increased exposure to surgical residents. Very few studies since then have surveyed the current status of robotics training in general surgery residency which is evolving rapidly, with the last survey of general surgery program directors dating back to 2002. Further investigations are needed to determine how different residency programs have adapted to incorporate robotic surgery, especially since the usage of robotics in surgery has increased. In addition, even with the incorporation of robotic surgery training, it is unclear how this training is evaluated, since each institution may have developed different methods to assess proficiency.

With robotic technology continuing to grow, minimally invasive surgery may be applicable to a more extensive array of procedures in the future. The objectives of this study were to (1) understand how much exposure general surgery residents currently have to robotic-assisted operations, (2) whether they have received any formal or informal training to robotic surgery, and (3) the effect of this platform on general surgical training.

## 2. Methods

### 2.1. Study Population

With Rutgers New Jersey Medical School Institutional Review Board approval, all study members were recruited from accredited general surgery training programs in the United States. The Accreditation Council for Graduate Medical Education website was used to identify 281 general surgery residency programs in October of 2017. Of those listed, 236 programs had email contact information for residency program directors.

### 2.2. Questionnaire

An online, Rutgers-associated survey tool, Qualtrics, was used to develop a 33-question survey, of which 5 were follow-up questions based on a skip pattern, in which the questions appeared based on the response to the certain questions (Appendix A). The purpose of this survey was to evaluate different perspectives of program directors on robotic surgery education. Specifically, participants were asked about their area of interest in general surgery, years of experience as a surgeon, and amount of experience with robotic surgery. Furthermore, the survey inquired about general aspects of their own surgical residency program, the volume of robotic surgery performed at their institution, and robotic surgery training in their residency program. Lastly, participants were requested to provide their view toward robotic surgery training for residents and achieving proficiency. Some survey questions were modified from previous studies that assessed the prevalence and application of robotic surgery education in residency programs and were therefore found to be still relevant [5, 9, 10].

An initial email was sent to the study population requesting their participation in December of 2017. The email contained an informed consent letter stating the objectives of the study and rights as a participant, an electronic link to the online questionnaire, and contact information of the principal investigator. An electronic reminder was sent to all participants three weeks later to maximize the response rate. The survey did not include any names or identifying information, ensuring privacy and confidentiality. The responses were password protected by the authors.

### 2.3. Statistical Analysis

The responses were automatically compiled descriptively as percentages for statistical analysis by the Qualtrics program. Basic statistics and table creation were performed with Microsoft Excel 2016.

## 3. Results

### 3.1. Demographics

The study sample included 20 total respondents; 18 respondents fully completed the survey, 1 respondent completed all 33 questions except the last 5 questions, and 1 respondent only completed the last 12 questions. The overall survey response rate was 8% (20 of 236 potential respondents). Of the participants who elected to answer, 10 were directors of a university residency program (52.63%), 2 of a university-affiliated residency program (10.53%), and 7 of a community/independent residency program (36.84%), as represented in Table 1. The majority of responding program directors were from medium-sized programs, defined in this study as 4–7 graduating residents per year. Specifically, these responses included 3 program directors of programs with 1–3 graduating residents per year (15.79%), 15 program directors of programs with 4–7 graduating residents per year (78.95%), and 1 program director of a program with 8+ graduating residents per year (5.26%). Next, there was a range of responses for number of attending general surgeons performing robotic surgery at each of the responding program directors' institution with 4 participants having 0–2 robotic surgeons (21.05%), 5 with 3–5 robotic surgeons (26.32%), 5 with 6–8 robotic surgeons (26.32%), and 5 with 8+ robotic surgeons (26.32%), as stated in Table 1. However, there was more variation between the number of cases performed each year for each respondent's institution: 6 reported their institutions performed less than 50 cases per year (33.33%), 4 reported their institutions performed 51–100 cases per year (22.22%), 3 reported their institutions performed 101–200 cases per year (16.67%), and 5 reported their institutions performed over 200 cases per year (27.78%).

The program directors' experiences and interests were also investigated (Table 2). When asked of their primary surgical interest, most program directors responded that they were interested in general surgery (6 of 19, 31.58%). There were 3 participants who responded in each of the specialties: surgical oncology, trauma surgery, and vascular surgery (15.79% each). Additionally, 2 participants specialized in colorectal surgery (10.53%) and another 2 were interested in minimally invasive surgery/laparoscopic surgery (10.53%). No program directors responded that they would be interested in any other field, including bariatric surgery, cardiothoracic surgery, dermatological surgery, endocrine surgery, neurosurgery, ophthalmology, oral and maxillofacial surgery, orthopedic surgery, otorhinolaryngology, pediatric surgery, plastic surgery, thoracic surgery, and urology. Additionally, most responding program directors did not currently use robotic surgery in their practice (73.68%). Of those who do, 3 participants have 0–3 years of experience in robotic surgery (60%) and 2 have for 4–6 years of experience (40%). Of the 5 who perform robotic surgery, 4 included the number of robotic cases they perform each month, varying from 1 to 6 cases (Table 2). Additionally, of the 14 respondents who do not currently use robotic surgery in their practice, only 2 have ever used robotic surgery in their practice (14.29%).

### 3.2. Current Robotic Surgery Training in General Surgery Residency

To understand the general trends in robotic surgery education and any differences between training, residency directors were questioned about their current training programs (Tables 3 and 4). The majority of responding program directors indicated that their institutions did have a formal clinical curriculum for robotic surgery training for their general surgery residents (73.68%, Table 3). Additionally, most have a formal simulation curriculum established for robotic surgery training (63.16%, Table 4). Regarding those without a formal simulation curriculum, most respondents perceived funding/cost (20.83%), faculty availability (20.83%), and access to simulators and facilities (20.83%) as the top barriers to including robotic simulation into their program (Table 4). Dedicated time for simulation (16.67%) and lack of facilities (16.67%) were also selected as barriers preventing the incorporation of a formal simulation curriculum. Only one participant found lacking national standards for robotic simulation as a barrier.

Most responding program directors stated that their residency programs incorporate residents' first exposure to robotic surgery in either postgraduate year 1 or 3 (41.11% and 31.58%, resp., Table 3). The postgraduate year (PGY) level at which residents begin to assist at the bedside of a robotic case varied, with a third of the programs beginning in PGY2 (33.33%, Table 4). As to when residents begin to perform as a console surgeon in a robotic case, many respondents reported that their programs allowed their residents to do so once they were a PGY4 (44.48%).

Currently, a majority of the participants with programs that included robotic surgery into their residency training, reported the use of a combination of teaching labs/simulation and operating room experience (84.21%, Table 3). Furthermore, most programs had a specific simulation training for residents in docking, instrument exchange, and console skills (73.68%, 82.35%, and 84.21%, resp.), but most did not have specific simulation training for specific robotic procedures such as cholecystectomy and hernia repair (57.89%, Table 4). To provide robotic surgery training to general surgery residents, most respondents indicated collaborating with industry (80%, Table 3).

Prior to allowing residents to assist in or perform a robotic surgery case, most respondents required residents to achieve proficiency on a robotic simulator (70%, Table 4). However, the majority did not require all graduating chief residents at their program to achieve competency in basic robotic operation prior to graduating (70%, Table 3). In these programs that did not require competency, most residents who do achieve competency in robotic surgery do so because of their interest in robotic surgery (78.57%). Unfortunately, for those graduating residents interested in further developing their robotic surgery skills, most program directors surveyed did not offer a minimally invasive and robotic surgery fellowship at their institution (89.47%).

### 3.3. Views on Robotic Surgery Education in General Surgery Residency

To understand future directions of robotic surgery training for general surgery residents, the current opinions on robotic surgery education were investigated (Tables 3 and 5). About two-thirds of the surveyed program directors believe that a formal robotics surgery curriculum should be incorporated in general surgery residency training (63.16%, Table 3). All respondents believe that residents should be first exposed to robotic surgery training within their first 3 years, with a majority thinking this should occur at the PGY1 level (55%). Interestingly, more than half of the participating program directors believe that more time should be dedicated to robotic surgery training during general surgery residency (52.63%, Table 5). More specifically, that more training time should be dedicated to robotic simulation training prior to console use in the operating room (84.21%).

As to the best method to deliver robotic training to general surgery residents, more than two-thirds (70%, Table 3) found it best to use a combination. The most common combination included using conference/didactic session, teaching labs/simulators, and the operating room. One participant suggested computer-based training, followed by simulation, then by bedside assisting, and finally console use. Others found that teaching labs/simulators are the best method to deliver robotic training (30%), as reported in Table 3. Consistent with current practice, many respondents still believe industry should play a role in this robotic training and simulation (63.16%).

With all the training and proficiencies required of graduating residents, most program directors do not find that proficiency in robotic surgery should be a required competency (Table 3). However, if proficiency/mastery needed to be determined, most participants would base it off of either a resident's level of involvement on a robotic surgery case or some sort of evaluation method of their robotic skills (40% for each method, Table 5), with one participant responding “Can the resident dock? Can the resident dissect? Can the resident maneuver the camera/change instruments? Can the resident sew simple versus complex cases?” as ways to assess robotic skills. Very few thought that the number of cases completed would be enough to determine proficiency of robotic surgery (20%). Lastly, a majority of program directors did not believe that a fellowship in robotic surgery should be required to safely perform robotic surgery cases (84.21%).

## 4. Discussion

This survey provided a unique opportunity to understand the perspectives of program directors on the implementation, usage, and assessment of robotic surgery training in general surgery residency programs. We found that the majority of responding program directors do not currently use robotic surgery in their practice, but their general surgery residency programs did have a formal robotic surgery curriculum as well as simulation training. For those that did not have simulation, the most common perceived barriers were cost, faculty availability, and access to simulators. With cost and access as two major barriers, industry may be able to play an important role in robotic surgery training. In fact, most respondents are of programs that currently collaborate with industry to provide robotic surgery training to residents and most responding program directors believe that they should continue to play a role in the future. This suggests that respondents acknowledge the importance of robotic surgery training, but its implementation may be impeded by financial or technical constraints. This is supported by the fact that a majority of the responding program directors believe a formal robotic surgery curriculum should be incorporated and that more time should be spent on robotic surgery training, especially with simulation prior to operating room experiences.

Responding program directors not only support increased emphasis on robotic surgery training but also advocate for early exposure. Program directors responded that, in both current practice and their perspective, residents have early exposure to robotic surgery, commonly in the first year. Program directors responded that the robotic surgery training commonly occurs as follows: introduction to robotic surgery during PGY1, achievement of proficiency via simulation prior to bedside assisting during PGY2, and then practicing and advancing until they can control the console, which usually occurs during PGY4. Despite this stepwise training, we found that chief residents are not required to attain competency in robotic surgery prior to graduation. Similarly, responding program directors did not feel this competency requirement should be implemented. Interestingly, a majority of the responding program directors believe that a fellowship is not necessary to safely perform robotic surgery cases. Essentially, general surgery residents are educated in robotic surgery throughout their residency, though it is not a required competency for graduation. Despite the lack of competency requirement, responding program directors feel the robotic surgery training during residency is sufficient for graduates to safely perform robotic surgery.

These results were supported by current literature. This survey revealed that most respondents' programs now provide exposure to robotics surgery during residency, which is supported by a more recent study surveying general surgery residents in 2013 [5]. Comparable to previous studies about robotics education, this study finds that there needs to be greater emphasis on robotics surgery education during residency [5, 10]. Furthermore, results from robotics education studies in other surgical specialties also support the results of this survey that there is a need to develop a structured curriculum in robotic surgery [11–14].

Similar to results of this study, recent studies also found that a majority of programs offer their residents early robotic training, with most beginning at the PGY1 levels [5, 10]. According to older studies, most programs used experiences assisting in the operating room as the main teaching method for residents, with minimal augmentation from teaching labs and conference/didactic session [5, 9, 10]. The results of this study do not support this result and, along with more recent studies, found that many programs have now switched to using a combination of teaching methods to educate residents in robotics [14, 15]. This difference may be due to the fact that robotics technology is rapidly developing, with more developed and accessible simulation training tools. By standardizing robotics education, general surgery residency programs can incorporate protected time toward developing this surgical method, which would ultimately improve surgical outcomes and better our patients.

This study had several limitations typical of survey-based studies. First, the response rate was low resulting in not enough responses to perform robust statistical analysis; therefore, only descriptive analysis was used. This low response rate makes it difficult to apply the conclusions of this paper to all general surgery residency programs and their program directors. Another limitation was that participation was voluntary, meaning it is possible that people who had strong opinions regarding robotic surgery were more likely to complete the survey, resulting in selection bias. Perhaps another method of distribution besides email would have increased the response rate, thereby reducing these limitations and enabling the application of these conclusions on a greater scale.

Overall, despite several limitations, this study has provided useful insights into the realities and program director perspectives of robotic surgery training. This information may contribute to the further incorporation of robotic training into the general surgery residency programs.

## 5. Conclusion

In conclusion, this study found that the majority of responding general surgery residency programs have robotic surgery training with the use of simulation. Respondents support this training, even though few perform robotic surgery themselves and in fact advocate for increased time and emphasis on robotic surgery training as well as early exposure for residents. This study has obtained valuable results regarding robotic surgery training and the perspectives of general surgery residency program directors which will contribute towards the continued progress of robotic surgery education in general surgery residency programs.

## Figures and Tables

**Table 1:** Institution demographics.

Question	Response
Residency program type (*N* = 19)	University (52.63%)
	University affiliated (10.53%)
	Community/independent (36.84%)

Number of graduating residents per year (*N* = 19)	1–3 residents (15.79%)
	4–7 residents (78.95%)
	8+ residents (5.26%)

Number of attending surgeons performing robotic surgery (*N* = 19)	0–2 surgeons (21.05%)
	3–5 surgeons (26.32%)
	6–8 surgeons (26.32%)
	9+ surgeons (26.32%)

Number of general surgery robotic cases each year (*N* = 18)	Less than 50 cases (33.33%)
	51–100 cases (22.22%)
	101–200 cases (16.67%)
	Over 200 cases (27.78%)

**Table 2:** Program director demographics.

Question	Response
Area of specialty interest or expertise within general surgery (*N* = 19)	Colorectal surgery (10.53%)
	General surgery (31.58%)
	Surgical oncology (15.79%)
	Trauma surgery (15.79%)
	Vascular surgery (15.79%)
	Other (10.53%, MIS/GI surgery/abdominal wall reconstruction, laparoscopic surgery)
	All others^*∗*^ (0%)

Number of years as a practicing surgery (*N* = 19)	0–4 years (0%)
	5–9 years (26.32%)
	10–14 years (15.79%)
	15–19 years (21.05%)
	20+ years (36.84%)

Use of robotic surgery in current practice (*N* = 19)	Yes (26.32%)
	No (73.68%)

If yes, amount of years using robotic surgery in practice (*N* = 5)	0–3 years (60%)
	4–6 years (40%)
	7–9 years (0%)
	10+ (0%)

If yes, number of cases performed each month (*N* = 4)	1 case
	2 cases
	1–3 cases
	6 cases

If no, any experience in robotic surgery in practice (*N* = 14)	Yes (14.29%)
	Never used robotic surgery (85.71%)

^*∗*^Other options included: Bariatric Surgery, Cardiothoracic Surgery, Dermatologic Surgery, Endocrine Surgery, Neurosurgery, Ophthalmology, Oral and Maxillofacial Surgery, Orthopedic Surgery, Otorhinolaryngology, Pediatric Surgery, Plastic Surgery, Thoracic Surgery, Urology.

**Table 3:** Comparison of current robotic surgery training to program director beliefs.

Question	Current practice	PG opinion
Is there/should there be a formal clinical curriculum for robotic surgery training of general surgery residents at your institution? (*N* = 19)	Yes (73.68%) No (26.32%)	Yes (63.16%) No (36.84%)

At which postgraduate year (PGY) level, do/should your residents first have exposure to robotic surgery? (*N* = 19, 20, resp.)	PGY1 (42.11%) PGY2 (10.53%) PGY3 (31.58%) PGY4 (10.53%) PGY5 (5.26%)	PGY1 (55%) PGY2 (15%) PGY3 (30%) PGY4 (0%) PGY5 (0%)

What is your program's current/the best method to deliver robotic surgery training during residency? (*N* = 19, 20, resp.)	Conference/didactic session (0%) Teaching labs/simulation (10.53%) Operating room experience (5.26%) Combination of the above (84.21%)	Conference/didactic session (0%) Teaching labs/simulation (30%) Operating room experience (0%) Combination of the above (70%)^*∗*^

Does/should your program collaborate with industry to provide robotic surgery training to residents? (*N* = 20, 19, resp.)	Yes (80%) No (20%)	Yes (63.16%) No (36.84%)

Do/should all graduating chief residents in your program achieve competency in this operation prior to graduation? (*N* = 20)	Yes (30%) No (70%)	Yes (35%) No (65%)

If not currently a competency, is resident achievement of competency based on resident's interest in robotic surgery? (*N* = 14)	Yes (78.57%) No (21.43%)	

^*∗*^By selecting “combination of the above,” respondents were requested to further elaborate. The responses included (*N* = 12) all 3 listed above (75%), computer based training, followed by simulation, followed by beside assist, finally console (8.33%), and simulation modulates then OR (16.67%).

**Table 4:** Current robotic surgery education method.

Question	Response		
Is there a formal simulation curriculum for robotic surgery training of general surgery residents at your institution? (*N* = 19)	Yes (63.16%) No (36.84%)		

What do you perceive as a barrier(s) to including robotic simulation in your program? (*N* = 24)	Funding/cost (20.83%) Faculty availability (20.83%) Dedicated time for simulation (16.67%) Lack of facilities (16.67%) Access to simulators and facilities (20.83%) Lack of scientific evidence (0%) Lack of national standards in robotic simulation (4.17%) Other (0%)		

At which postgraduate year (PGY) level do most residents in your program begin to assist at the bedside of a robotic case? (*N* = 18)	PGY1 (22.22%) PGY2 (33.33%) PGY3 (27.78%) PGY4 (11.11%) PGY5 (5.56%)		

At which postgraduate year (PGY) level do most residents in your program begin to perform as a console surgeon in a robotic case? (*N* = 18)	PGY1 (5.56%) PGY2 (0%) PGY3 (27.78%) PGY4 (44.44%) PGY5 (22.22%)		

Does your program have specific simulation training for residents in any of the following tasks:	Docking (*N* = 19)	Yes (73.68%)	No (26.32%)
	Instrument exchange (*N* = 17)	Yes (82.35%)	No (17.65%)
	Console skills (*N* = 19)	Yes (84.21%)	No (15.79%)
	Specific robotic procedures [cholecystectomy, hernia repair, etc.] (*N* = 19)	Yes (42.11%)	No (57.89%)

Does your program require residents to achieve proficiency on a robotic simulator prior to assisting in, or performing, a robotic surgery case? (*N* = 20)	Yes (70%) No (20%)		

Does your institution offer a minimally invasive and robotic surgery fellowship? (*N* = 19)	Yes (10.53%) No (89.47%)		

**Table 5:** Views on robotic surgery education method.

Question	Response
Should more time be dedicated to robotic surgery training during general surgery residency? (*N* = 19)	Yes (52.63%) No (47.37%)

Should more time be dedicated to robotic simulation training prior to resident console use in the operating room? (*N* = 19)	Yes (84.21%) No (15.79%)

How should proficiency/mastery of robotic surgery be determined? (*N* = 20)	Number of cases completed (20%%) Level of involvement on RS cases (40%) Other (40%)^*∗∗*^

Do you believe a fellowship in robotic surgery should be required to safely perform robotic surgery cases? (*N* = 19)	Yes (15.79%) No (84.21%)

^*∗∗*^By selecting “other,” respondents were requested to further elaborate. The responses included (*N* = 6) measured performance of surgeons with excellent robotic surgery outcomes, validated metrics, a combination of standardized evaluation, competency evaluations, and procedures, PD evaluation, and EPA's such as -- can the resident dock/can the resident dissect/can the resident maneuver the camera/change. Instruments/can the resident sew simple versus complex cases, and OSATs.

## Supplementary Materials

Supplementary Materials Appendix A contains our 33-question survey. Both multiple choice and open-ended questions were used. Five questions were follow-up questions based on a skip pattern, in which the questions appeared based on the response to certain questions.
