# Supplementary material for: Residency Training in Robotic General Surgery: A Survey of Program Directors
Source: Minim Invasive Surg. 2018 May 8;2018:8464298. doi: 10.1155/2018/8464298 (PMC5964613; doi:10.1155/2018/8464298)
Supplement: Supplementary Materials — Appendix A contains our 33-question survey. Both multiple choice and open-ended questions were used. Five questions were follow-up questions based on a skip pattern, in which the questions appeared based on the response to certain questions. [file 8464298.f1.docx]

**Appendix A**

Please select the appropriate response for each of the following:

1. Residency Program Type:
   1. University
   2. University-Affiliated
   3. Community/Independent
2. Number of graduating residents per year:
   1. 1-3
   2. 4-7
   3. 8+
3. Area of specialty interest or expertise within general surgery:
   1. Bariatric Surgery
   2. Cardiothoracic Surgery
   3. Colorectal Surgery
   4. Dermatologic Surgery
   5. Endocrine Surgery
   6. General Surgery
   7. Neurosurgery
   8. Ophthalmology
   9. Oral and Maxillofacial Surgery
   10. Orthopedic Surgery
   11. Otorhinolaryngology
   12. Pediatric Surgery
   13. Plastic Surgery
   14. Surgical Oncology
   15. Thoracic Surgery
   16. Trauma Surgery
   17. Urology
   18. Vascular Surgery
   19. Other
4. If you selected other, please specify your area of specialty interest or expertise within general surgery:

__________________________________________

1. How many years have you been a practicing surgeon?
   1. 0 – 4
   2. 5 – 9
   3. 10 – 14
   4. 15 – 19
   5. 20 +
2. Do you currently use robotic surgery in your practice?
   1. Yes
      1. For how many years have you used robotic surgery in your practice?
         1. 0 – 3
         2. 4 – 6
         3. 7 – 9
         4. 10+
      2. About how many robotic cases do you take part in each month? __________________ / month
   2. No
      1. Have you ever used robotic surgery in your practice?
         1. Yes
         2. No, never used robotic surgery

In this section, we would like to know more about robotic surgery at your general surgery residency program:

1. How many attending general surgeons perform robotic surgery at your institution?
   1. 0 - 2
   2. 3 - 5
   3. 6 - 8
   4. 9+
2. Roughly, how many robotic general surgery cases does your department perform each year?
   1. Less than 50
   2. 51-100
   3. 101-200
   4. Over 200
3. Does your institution offer a minimally invasive and robotic surgery fellowship?
   1. Yes
   2. No
4. Is there a formal clinical curriculum for robotic surgery training of general surgery residents at your institution?
   1. Yes
   2. No
5. Is there a formal simulation curriculum for robotic surgery training of general surgery residents at your institution?
   1. Yes
   2. No
      1. If no, what do you perceive as a barrier(s) to including robotic simulation in your program? (Choose all that apply.)
         1. Funding/Cost
         2. Faculty availability
         3. Dedicated time for simulation
         4. Lack of facilities
         5. Access to simulators and facilities
         6. Lack of scientific evidence
         7. Lack of national standards in robotic simulation
         8. Other ______________________
6. At which post-graduate year (PGY) level, are your residents first exposed to robotic surgery?
   1. PGY1
   2. PGY2
   3. PGY3
   4. PGY4
   5. PGY5
7. What is your program’s current method to deliver robotic surgery training during residency?
   1. Conference/didactic session
   2. Teaching labs/simulation
   3. Operating room experience
   4. A combination of the above
   5. None of the above
8. Do you have specific simulation training for residents in any of the following robotic tasks:
   1. Docking: Yes/No
   2. Instrument exchange: Yes/No
   3. Console skills: Yes/No
   4. Specific robotic procedures (cholecystectomy, hernia repair, etc): Yes/No
9. At which post-graduate year (PGY) level do most residents in your program begin to assist at the bedside of a robotic case?
   1. PGY1
   2. PGY2
   3. PGY3
   4. PGY4
   5. PGY5
   6. N/A
10. At which post-graduate year (PGY) level do most residents in your program begin to perform as a console surgeon in a robotic case?
    1. PGY1
    2. PGY2
    3. PGY3
    4. PGY4
    5. PGY5
    6. N/A
11. Do you require residents to achieve proficiency on a robotic simulator prior to assisting in, or performing, a robotic surgery case?
    1. Yes
    2. No
12. Does your program collaborate with industry to provide robotic surgery training to residents?
    1. Yes
    2. No
13. Focusing specifically on a basic robotic operation (e.g., cholecystectomy), do ALL graduating chief residents in your program achieve competency in this operation prior to graduation?
    1. Yes
    2. No
       1. If no, is resident achievement of competency based on resident interest in robotic surgery?
          1. Yes
          2. No

In this section, we would like to understand your view on robotic surgery training for general surgery residents:

1. At which post-graduate year (PGY) level, should residents be first exposed to robotic surgery training?
   1. PGY1
   2. PGY2
   3. PGY3
   4. PGY4
   5. PGY5
2. What is the best method to deliver robotic surgery training during residency, in your opinion?
   1. Conference/didactic session
   2. Teaching labs/simulators
   3. Operating room
   4. Combination; specify: _______________________
3. Do you believe that proficiency in robotic surgery should be a required competency for residency?
4. Yes
5. No
6. How should proficiency/mastery of robotic surgery be determined?
   1. Number of cases completed
   2. Level of involvement on a robotic surgery case
   3. Other: ______________________________________________
7. Should more time be dedicated to robotic surgery training during general surgery residency?
   1. Yes
   2. No
8. Do you believe a formal robotics surgery curriculum should be incorporated in general surgery residency training?
   1. Yes
   2. No
9. Should more time be dedicated to robotic simulation training prior to resident console use in the operating room?
   1. Yes
   2. No
10. Do you believe industry should play a role in the training of residents in robotic surgery?
    1. Yes
    2. No
11. Do you believe, a fellowship in robotic surgery should be required to safely perform robotic surgery cases?
    1. Yes
    2. No
